# POSITIVE EMOTION EXPRESSION AND ADAPTIVE COPING BEHAVIORS FOR ADULT CHILDREN OF PARENTS WITH MEMORY LOSS

**DOI:** 10.1093/geroni/igac059.2796

**Published:** 2022-12-20

**Authors:** Dustin Gad, Amanda Piechota, Joan Monin

**Affiliations:** Yale School of Public Health, New Haven, Connecticut, United States; Yale School of Public Health, New Haven, Connecticut, United States; Yale School of Public Health, New Haven, Connecticut, United States

## Abstract

The broaden-and-build theory suggests that experiences of positive emotion may lead to enhanced utilization of adaptive coping strategies, and a decrease in maladaptive coping strategies (Gloria & Steinhardt, 2016). This relationship between positive emotion and adaptive coping has yet to be studied directly in a sample of adult child caregivers of a parent with memory loss. As part of a larger study investigating relationship dynamics between adult children and their parents with memory loss, adult children 18 years of age and older (n= 67) responded to self-report surveys and engaged in a 6 minute, video recorded, positive interaction session, playing “name that tune” with each other. The session was observationally coded by two coders for “enjoyment/enthusiasm/fun” (k= .516), “laughter” (k= .631), and “positive affect displayed towards partner” (k=.464), using a reliable and valid support-seeking and caregiving behavior coding system (Collins & Feeney, 2000). Spearman’s rank correlations between these behavior codes and the self-reported Brief COPE Inventory (BCI) scores suggested positive correlations between “laughter” and the emotional support subscale of the BCI (rs=.259, p= .034), and between “positive affect displayed towards partner” and the venting subscale of the BCI (rs= .256, p= .036). These findings suggest that the expression of positive emotions in the caregiving process might yield important psychological benefits to the caregiver, through increased utilization of specific coping mechanisms. More research in this area is needed to determine whether positive emotion expression is associated with the use of adaptive coping more so than maladaptive coping mechanisms in this population.

